# IGHV mutational status of nodal marginal zone lymphoma by NGS reveals distinct pathogenic pathways with different prognostic implications

**DOI:** 10.1007/s00428-019-02712-8

**Published:** 2019-12-04

**Authors:** Massimo Granai, Teresa Amato, Arianna Di Napoli, Raffaella Santi, Federica Vergoni, Gioia Di Stefano, Virginia Mancini, Sofya Kovalchuk, Emanuele Cencini, Alberto Giulio Carta, Sara Aversa, Marita Ziepert, Gabriele Cevenini, Stefano Lazzi, Lorenzo Leoncini, Cristiana Bellan

**Affiliations:** 1 Department of Medical Biotechnologies, Anatomic Pathology Division, University of Siena, Via delle Scotte, 6, 53100 Siena, Italy; 2 Department of Clinical and Molecular Medicine, Pathology Unit, University of Rome “La Sapienza”, Rome, Italy; 3 Florence Pathology Unit, Careggi University Hospital, Florence, Italy; 4 Florence Hematology Unit, University of Florence, Florence, Italy; 5 Department of Hematology, University of Siena, Siena, Italy; 6 Institute for Medical Informatics, Statistics and Epidemiology, University of Leipzig, Leipzig, Germany

**Keywords:** BCR, Nodal marginal zone lymphomas, NGS, Clonality analysis

## Abstract

The precise B cell of origin and molecular pathogenesis of nodal marginal zone lymphoma (NMZL) remain poorly defined. To date, due to the rarity of NMZL, the vast majority of already-published studies have been conducted on a limited number of samples and the technical approach to analyze the immunoglobulin genes was of amplifying rearranged variable region genes with the classical direct sequencing of the PCR products followed by cloning. Here, we studied the B cell Ig heavy-chain repertoires by next-generation sequencing (NGS) in 30 NMZL cases. Most of the cases were mutated (20/28; 71.5%) with homologies to the respective germ line genes ranging from 85 to 97, 83%, whereas 8/28 (28.5%) were unmutated. In addition, our results show that NMZL cases have a biased usage of specific immunoglobulin heavy-chain variable (IGHV) region genes. Moreover, we documented intraclonal diversity in all (100%) of the mutated cases and ongoing somatic hypermutations (SHM) have been confirmed by hundreds of reads. We analyzed the mutational pattern to detect and quantify antigen selection pressure and we found a positive selection in 4 cases, whereas in the remaining cases there was an unspecific stimulation. Finally, the disease-specific survival and the progression-free survival were significantly different between cases with mutated and unmutated IGHV genes, pointing out mutational status as a possible new biomarker in NMZL.

**Electronic supplementary material:**

The online version of this article (10.1007/s00428-019-02712-8) contains supplementary material, which is available to authorized users.

## Introduction

Nodal marginal zone lymphomas (NMZL) represent one of three recognized entities within the category of marginal zone lymphomas (MZL), along with splenic marginal zone lymphomas (SMZL) and extranodal marginal zone lymphomas (ENMZL), with the latter tumors also known as mucosa-associated lymphoid tissue (MALT) lymphomas. NMZL, SMZL, and MALT all belong to the category of indolent small B cell lymphomas [1]. Although NMZL shares many histologic and immunologic features with extranodal MZL of MALT type, clinical characteristics, natural history, and prognosis suggest that nodal MZL should be considered a distinct entity [2].

However, lack of typical markers and absence of a clear consensus for its molecular pathogenesis make the diagnosis of nodal marginal zone lymphoma (NMZL) a problematic subject [3]. Yet, the precise B cell of origin of NMZL remains poorly defined [4].

A B cell undergoes germinal center (GC) reaction in response to antigen stimulation, resulting in the generation of a memory B cell with a high specificity and affinity. At the gene level, memory B cells are characterized by somatic mutation (SM) in their rearranged immunoglobulin (Ig) heavy-chain variable (VH) genes [5]. Somatic mutation studies of SMZL and ENMZ have shown that in the great majority of the cases, the tumor cells are of a post-GC, memory B cell derivation, displaying a mutational pattern indicative of positive antigen selection [6].

Because of the rarity of NMZL, it is hard to obtain large study groups, and in all previous studies, the technical approach of amplifying rearranged variable region genes was the classical sequencing methods, i.e., direct sequencing of the PCR products followed by cloning. However, this approach is based on the analysis of limited number of clones that could not be representative for the real intraclonal heterogeneity.

The quantitative nature of next-generation sequencing (NGS) data allows for higher resolution of the subclonal architecture and can be used to decipher mutational signatures and, thus offering a dynamic mechanism for the mutations found in the sample [7].

Here, we studied the B cell Ig heavy-chain repertoires to characterize the diversity of the heavy-chain CDR3 region and the constituent V, D, and J segments that comprise it, in 30 NMZL cases to acquire insight into the nature of its cell of origin and to identify mutation patterns reminiscent of antigen selection processes. Our results show that NMZL cells have a biased usage of IGHV genes in favor of specific segments. We also shed light on the role of antigenic stimulation in the aetiology of NMZL and in the maintenance of BCR integrity. In addition, the postulated normal counterpart of this lymphoma consists of specific B lymphocyte subsets, with cases carrying unmutated and mutated IGHV genes which impact the clinical outcome as observed in chronic B cell leukemia (B-CLL) and other small B cell lymphomas.

## Materials and methods

### Patients and tissues samples

Thirty NMZL formalin-fixed paraffin-embedded (FFPE) cases were selected from the files of the Department of Medical Biotechnologies, University of Siena; Pathology Unit, Careggi University Hospital, Florence; and Department of Pathology, La Sapienza University, Rome. In all the cases, the diagnosis of NMZL was performed primarily on lymph node localization in the absence of previous or concurrent involvement of any extranodal site, with the exception of bone marrow. All the cases were reviewed by expert hematopathologists by morphological and immunohistochemical criteria according to WHO classification. In addition, to rule out a possible misdiagnosis of lymphoplasmacytic lymphoma, all cases were analyzed for MYD88 L265P mutation and two cases carrying the mutation of this gene were excluded from the study [8, 9]. As further validation of NMZL diagnosis, we also demonstrated the absence of glycosylation motifs in the VDJ regions of all the analyzed cases, hence excluding concealed follicular lymphomas [10].

### PCR amplification and high-throughput sequencing by Roche 454 GS Junior instrument

Genomic DNA was extracted from 5 to 10 μm of FFPE tissue using a DNA extractor (MagCore NucleicAcid Extractor, RBC Bioscience, Taiwan) and MagCore Genomic DNA FFPE One-Step Kit, following the manufacturer’s recommendations. Genomic DNA quality was assessed using BIOMED-2 control gene PCR protocol and samples with a DNA product size of ≥ 300 base pairs (bp) were analyzed [11]. Before initiating VDJ gene rearrangement analysis by HTS, all cases were analyzed to evaluate clonality according to the BIOMED-2 protocol [11]. NGS analysis was performed on 454 GS Junior system (Roche) previously described [12]. Data analysis was performed using the Roche (Basel, Switzerland) proprietary software package for the 454 GS Junior system (Roche). Image acquisition, image processing, and signal processing were performed during the run.

### Bioinformatical analysis

The bioinformatical analysis was performed by using the 454 GS Junior system, as previously described [12].

### Sequence data analysis

To determine the IGHV, IGHD, and IGHJ gene usage and the mutational status of each IGHV gene, sequences were submitted to the international ImmunoGeneTics (IMGT, Montpellier, France) database [13, 14] and aligned to the closest matching germ line gene by using the IMGT/V-QUEST and IMGT/Junction Analysis software [15, 16], as previously described [12].

### Clustering of VH CDR3 sequences

The length of the VH CDR3 of the immunoglobulin heavy-chain gene rearrangement was computed using the IMGT database starting from the first codon after the conserved cysteine up to the position preceding the conserved tryptophan of the JH gene segment, as previously described [17–19].

### Antigen selection

We used a recently published tool known as BASELINE (i.e., Bayesian estimation of antigen-driven selection; http://clip.med.yale.edu/selection) to detect and quantify antigen selection in individual or multiple sequences based on mutational patterns, normalized to germ line sequences, and provided a visual representation of differences in selective pressure [20, 21]. Clonally related sequences and productive heavy-chain V-region sequences (CDR1-FWR2-CDR2-FWR3) were analyzed using BASELINE version 1.3 (01/30/2014).

Typical antigen-driven activation results in positive selection in the complementary-determined regions (CDRs), which directly interact with antigen, and negative selection in the framework regions (FRs), which are more important for structural integrity. Patterns of selective pressure contrary to this model indicate non-specific activation [22].

### Statistical analysis

A multivariate analysis based on Cox’s proportional hazards regression was performed to verify the potential relationship between survival of the patients, mutational status, and critical clinical parameters (e.g., age, ECOG, LDH, stage, and therapeutic regimen) [23].

Survival curves were plotted using the Kaplan-Meier method and were compared using log-rank test. According to Cheson et al., overall survival (OS) was defined as the time from diagnosis to death; patients who remained alive were censored at the last date of follow-up [24]. Progression-free survival (PFS) was defined as the time from diagnosis to the date of first documented recurrence. Disease-specific (or disease-related) survival (DSS) was calculated from the date of diagnosis until the patient’s death due to the NMZL. Statistical analysis was performed using SPSS software version 20.0 [25]. For all the tests, *p* < 0.05 (two-sided) was considered statistically significant.

## Results

### Histopathological and immunophenotypic features

Most of the cases were characterized by a parafollicular and/or interfollicular infiltrate of neoplastic cells effacing the lymph node architecture and, to a considerably lesser extent, regressed residual lymphoid follicles, lacking well-formed germinal centers with attenuated mantle cuffs. The neoplastic cells were heterogeneous in appearance with monocytoid, centrocyte-like blastic and plasmacytoid features. All of the cases expressed pan-B cell markers (CD20, PAX5). Moreover, CD23 was also negative in the vast majority of the cases (21/28; 75%). CD21 showed a disrupted and expanded residual meshwork. All of the cases were negative for CD5 and cyclin D1. Germinal center markers (CD10, BCL6) were likewise negative. IgD IHC was also negative where performed. Conversely, IgM IHC, when available, was positive.

### High-throughput sequencing analysis of IGHV gene repertoire in NMZL

A total of 180,050 reads were generated. During the platform-specific processing, 70,904 reads failed the filtering process owing to missing or incomplete barcodes. For our 28 samples, 109,146 reads were obtained as final 454 output with an average depth of 2831 reads, with a minimum and maximum depth of 808 and 16,114 reads respectively. Unproductive rearrangements were excluded from analysis.

The IGHV, IGHD, and IGHJ gene and allele usage were obtained using the statistical analysis of IMGT/HighV-QUEST available online. This analysis is performed automatically on the “1 copy”| “single allele” (for V, D, and J) category. All the 28 cases were clonal on NGS using the criterion that a clonal cluster(s) must beat least fourfold more abundant than the largest clonotype of the background [12, 26]. In particular, the presumed monoclonal clusters, represented from 20 to 99% of the total reads, confirm the results of GeneScan profiles ranging from clonal to clonal with polyclonal background according to BIOMED-2 criteria. When all the sequences were aligned with IMGT tools for nucleotide analysis of immunoglobulin (IG), polymorphisms, and IG mutations, clusters showing identical IGHV, IGHD, and IGHJ usage and CDR3 regions as the presumed monoclonal clusters were detected. All the results representative of clonotypes AA (amino acid) identified by NGS were overlapped and confirmed with the results obtained by Sanger sequencing.

**Fig. 1:**
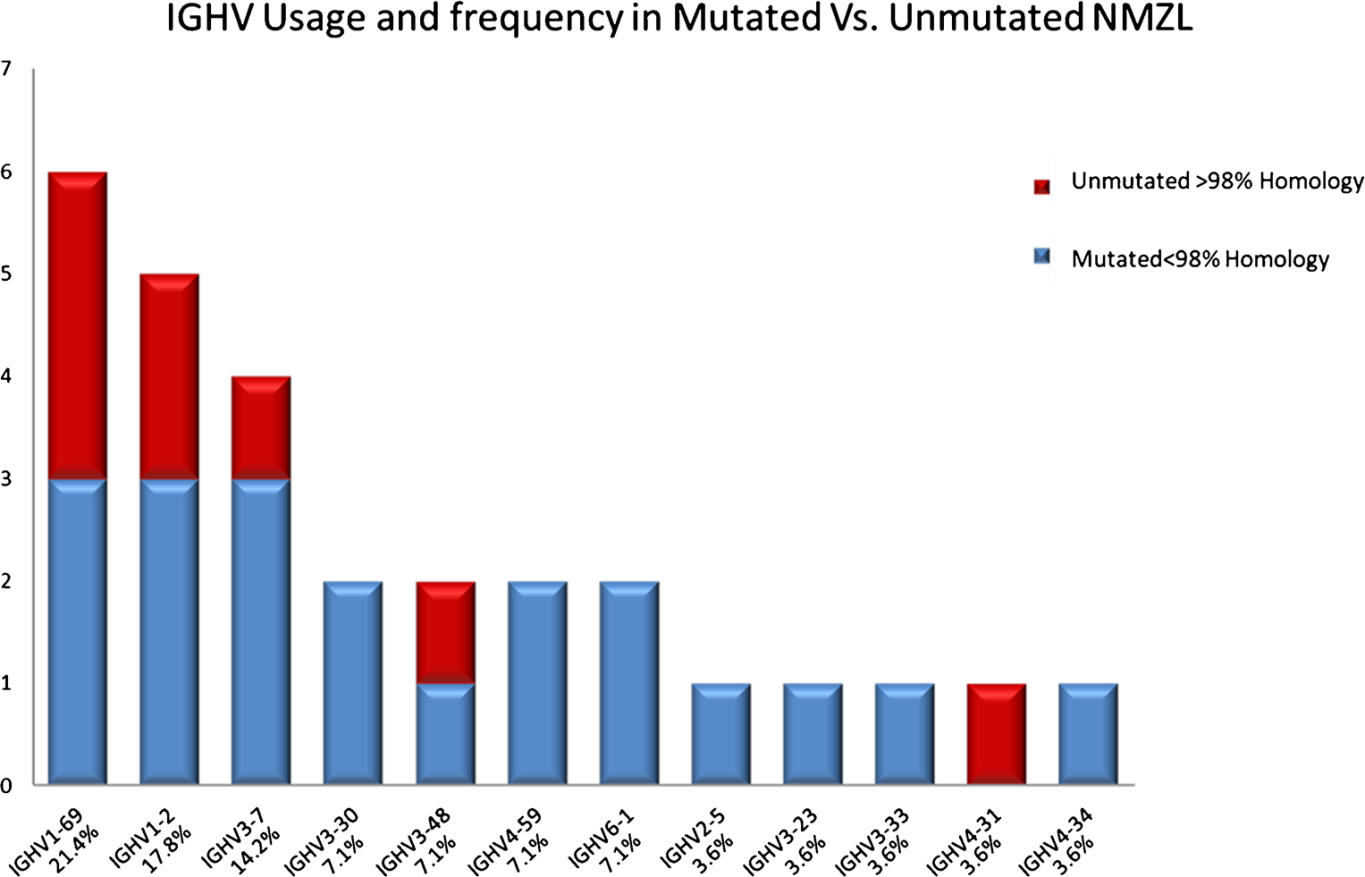
Gene usage in mutated vs unmutated NMZL. 6 out of 28 cases (21.4%) utilized *VH1-69* gene (3 mutated; 3 unmutated), 5 out of 28 (17.8%) were most homologous to a *VH1-2* gene segment (3 mutated; 2 unmutated), and 4 of 28 (14.2%) were most homologous to a *VH3-7* gene segment (3 mutated; 1 unmutated) while the remaining 13 were most closely related to different VH genes from the VH2 family (*VH2-5*), VH3 family( *VH3-23*, *VH3-30*, *VH3-33*, *VH3-48*), VH4 family (*VH4-31*, *VH4-34*, *VH4-59*), and VH6 family (*VH6-1*)

Most of the cases were mutated (20/28; 71.5%) (M-NMZL) with homologies to the respective germ line genes ranging from 85 to 97, 83%, whereas 8/28 (28.5%) were unmutated (U-NMZL) (Fig. 1).

**Fig. 2:**
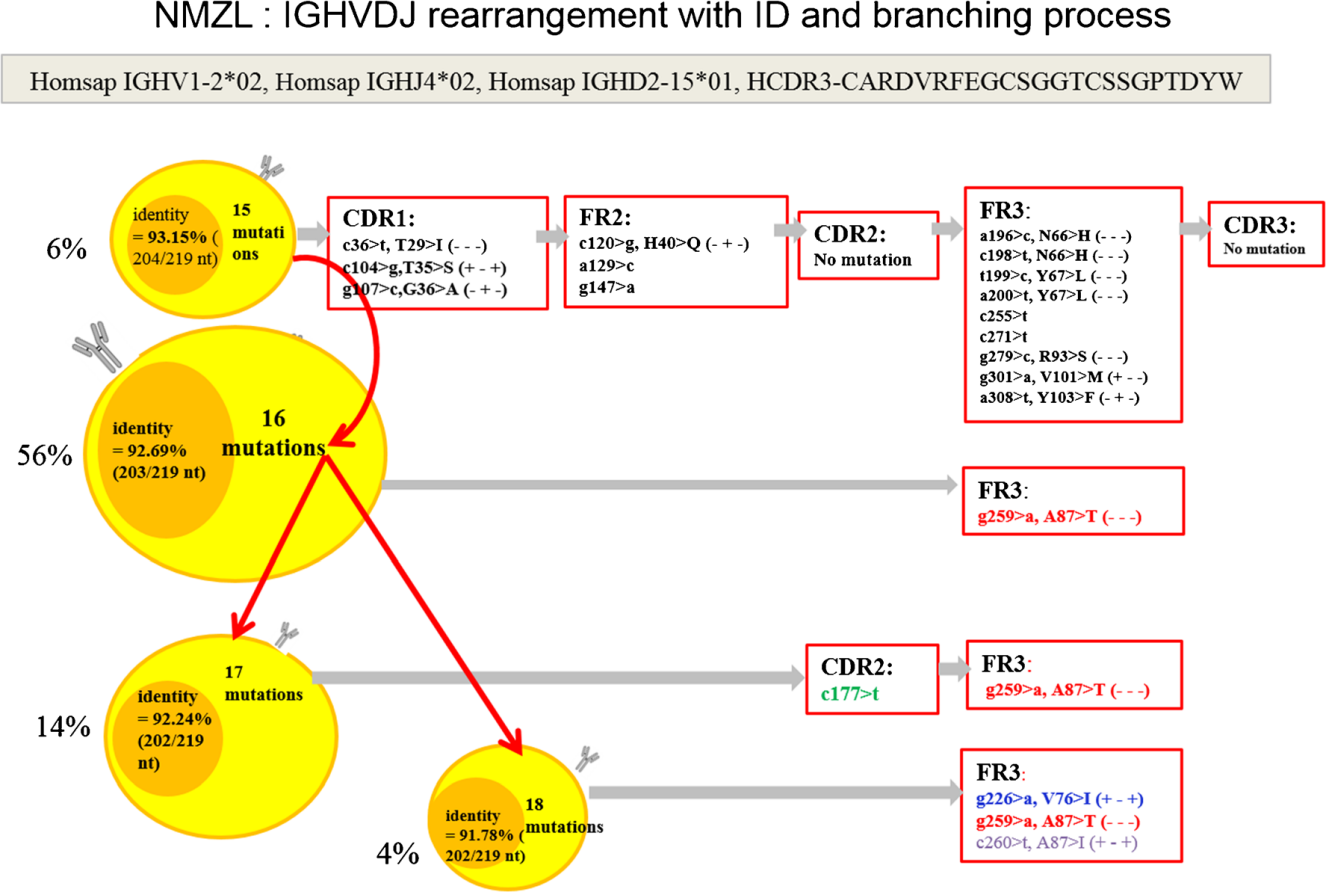
Example of branching of a lymphoma clone. The four different clusters represented show identical IGHV, IGHD, and IGHJ usage and related CDR3 regions but exhibit different somatic mutations. The mutations indicated in black color in the box are common mutations, while those in red, blue, and green are ongoing mutations. The dominant clone is confirmed by 56% of the sequences of clonotype AA and carried 16 mutations. The other three minor clones are represented by 6%, 14%, and 4% of the sequences of the clonotype AA. Respectively, all of the clones shared common mutations of the first clone (6% of sequences)

We demonstrated intraclonal diversity (ID) in all (100%) the patients with a mutated IGHV; ongoing SHM have been confirmed by hundreds of reads. Detailed results are reported in Supplementary Table 1. Subclones have been identified with a mean of 2–6 subclones per case. Figure 2 illustrates an example of the branching of the lymphoma clone and shows that distinct subclones evolved along similar, although separate pathways.

Nineteen productive heavy-chain V-region clonally related sequences were evaluated for selection pressure (Supplementary Table 1). The range of mutations was 30–7 with a V-region germ line identity% range 85.00–96.85. The BASELINE method found positive selection in the CDRs and negative selection in the FRWS of the heavy chains in 4 patients indicating selective pressure by antigens. In the remaining cases, we observed only a negative selection in the FRWS indicating a non-specific activation by the antigen/antigens to maintain the structural conservation and integrity of BCR [27] (Supplementary Table 2; Supplementary Figure 1).

### Analysis of IGHV Gene Usage

Clonal and in-frame VH gene sequences from NMZL cases derived from VH1, VH2, VH3, VH4, and VH6 families and were further stratified according to the mutational status. In particular, 6 out of 28 cases (21.4%) utilized the same *VH1-69* gene (3 mutated; 3 unmutated), 5 out of 28 (17.8%) were most homologous to a *VH1-2* gene segment (3 mutated; 2 unmutated), and 4 of 28 (14.2%) were most homologous to a *VH3-7* gene segment (3 mutated; 1 unmutated) while the remaining 13 were most closely related to different VH genes from the VH2 family (*VH2-5*), VH3 family (*VH3-23*, *VH3-30*, *VH3-33*, *VH3-48*), VH4 family (*VH4-31*, *VH4-34*, *VH4-59*), and VH6 family (*VH6-1*) (Fig. 1).

In addition, we also determined the IGHD and IGHJ genes used in IGHV-D-J sequences analyzed. The IGHD genes used were IGHD3 (35.7%), IGHD1 (21.4%), and IGHD2 (14.2%) and several others to a much lower extent, including IGHD4, IGHD5, and IGHD6. The IGHJ gene usage in all cases showed that IGHJ4 (46.4%) was used the most, followed by IGHJ6 (35.7%), IGHJ3 (3.57%), and IGHJ5 (7.1%).

The average VH CDR3 length of NMZL cells was 15, 7 AA ranging from 8 to 23 residues. In addition, we compared the CDR3 regions of the NMZL cases to previously published cases of CLL and SMZL that used the same VH region, and they differ in length and AA composition [6, 17, 18].

### Pattern of progression and survival

After a median follow-up of 5 years, no patient had developed splenic or MALT involvement during the course of disease. Additional clinical information is summarized in Table 1.

**Table 1:** Summarized clinical features with therapy of 28 NMZL cases

	*N*/tot	Percent
Clinical features		
Male	15/28	53
Median age (years)	67	
Age > 60 (years)	22/28	78
Peripheral lymph nodes	20/28	71
Abdominal/thoracic lymph nodes	8/28	29
Bone marrow involvement	11/28	40
Liver involvement	0/28	0
Spleen involvement	0/28	0
Pleural localization	0/28	0
LDH normal	23/28	82
ECOG ≥ 2	1/28	3
Global median OS (months)	66	(95% CI 52.9–79.0)
Treatments		
Watch and wait	6/28	21
R-Bendamustine	18/28	3
R-Leukeran, R-FC	4/28	14

*R-FC* rituximab-fludarabine-cyclophosphamide

At the time of the analysis, 12 patients were deceased. Death related to lymphoma occurred in 5/28 patients. Relapse of disease occurred in 10 patients. Global median time of overall survival (OS) was 66 months (95% CI 52.9–79.0).

**Fig. 3:**
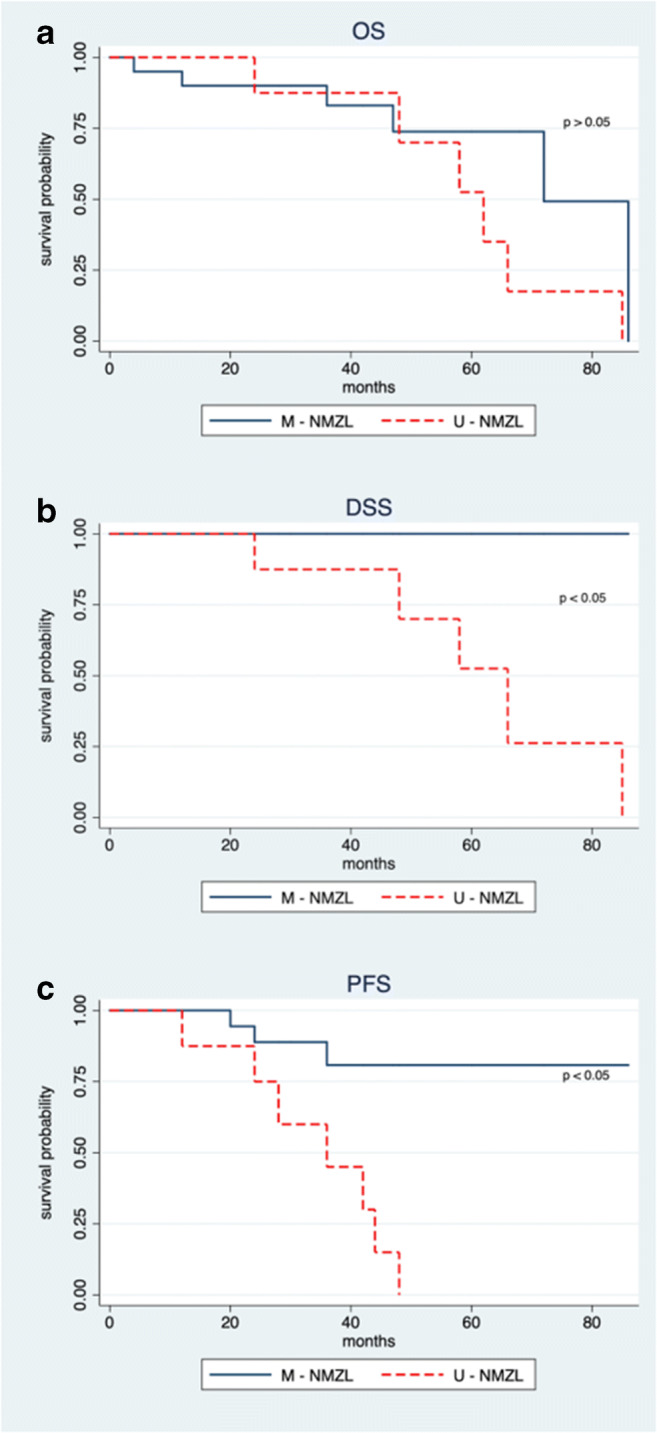
Survival analysis for NMZL according to the mutational status. Kaplan-Meier survival curves for overall survival in U-NMZL and M-NMZL patients (*p* = 0.18) (**a**). Kaplan-Meier survival curves for disease-specific survival in U-NMZL and M-NMZL patients (*p* < 0.01) (**b**). Kaplan-Meier survival curves for progression-free survival in U-NMZL and M-NMZL patients (*p* < 0.01) (**c**)

Despite the low sample size, we applied the multivariate Cox survival analysis. At univariate analysis, ECOG (*p* 0.035), IGHV status (*p* 0.005) with LDH (*p* 0.026), and IGHV status (*p* 0.0002) were respectively significant for OS, DSS, and PFS (Supplementary Table 3). However, elevated LDH and ECOG were infrequent (18% and 3%, respectively). Considering all the bivariate combinations, only the mutational status remains significant in DSS and PFS analyses. Cox models with higher dimensionality were completely not statistically significant. Therefore, the mutational status showed to be an independent factor affecting survival and consequently clinical variables did not significantly affect survival or acted as adjustment factors changing the mutational status contribution. Accordingly, patients were stratified on the grounds of IGHV mutational status. A median time of overall survival of 62 months (95% CI 46.5–77.5) and 72 months (95% CI 49.6–94.4) was shown for U-NMZL and M-NMZL patients, respectively. However, Kaplan-Meier survival curves for OS (Fig. 3a) showed a non-statistically significant difference between unmutated and mutated patients (*p* = 0.18). Interestingly, disease-specific survival (Fig. 3b) and progression-free survival (Fig. 3c) both exhibited a high significant difference between the two groups (*p* < 0.01). In particular, for the unmutated patients, the median times of DSS and PFS were 66 months (95% CI 48.9–83.1) and 36 months (95% CI 16.3–55.7), respectively.

## Discussion

Analyses of antigen-receptor genes in human lymphoma represent a useful tool in understanding their pathogenesis and clonal history [7].

Somatic hypermutations seem to be restricted to B cells proliferating within the microenvironment of the germinal center (GC). As a consequence, the presence of somatic mutations in the variable region of the rearranged immunoglobulin genes is actually considered the hallmark of B cells that have participated in a GC reaction [28]. Moreover, the pattern of the distribution of somatic mutations and a preferential usage of immunoglobulin variable, diversity, and joining segments may reveal a role of antigens in driving B cell proliferation. Clustering of nucleotide mutations leading to an amino acid substitution in the CDRs of VH segments is considered to indicate that the hypermutation process is driven by an antigen [22].

Here we show that in NMZL cases, the VH1 family genes were significantly overrepresented compared with transitional B cells, naive B cells, and IgM memory B cells [29]. In particular, our data are in accordance with previous studies which showed a biased usage of the *IGHV* genes in favor of *IGHV-1-69* (6 out of 28 cases; 21.4%) [30]. Conversely, we found an overrepresentation of *VH1-2* gene in our cohort of cases (5 out of 28; 17.8%) which has been found mainly in SMZL [30]. *VH1-2* is known to react with antigen exposed on apoptotic cells, suggesting that at least a subset of NMZL may arise from a self-antigen antibody producing B cell [6]. Furthermore, *VH1-69* gene segment is the most used in hepatitis C virus–positive NMZL [31]. However, the use of *VH1-69* gene in our series is not restricted to hepatitis C virus infection. In fact, *VH1-69* utilizing antibodies are also found in protective antibody responses to additional viral pathogens such as influenza infection, respiratory syncytial virus infection, and HIV-1 [32]. On the other hand, we could not confirm in this series an overrepresentation of *VH4-34*, as reported in previous studies [30, 33].

In line with previous studies, 20 out of 28 cases (71.5%) carried SHM in their immunoglobulin genes [30]. We confirmed ID in all the mutated NMZL subgroup of patients analyzed, supporting the notion that the SHM mechanism remains active post-transformation and outside the germinal centers, further diversifying the clonotypic IG receptors. Therefore, the finding of ongoing mutations as indicated by intraclonal variations in NMZL provides the genetic evidence that the tumor responds to antigen stimulation, which may play an important role in its clonal expansion [34]. Several other studies have also demonstrated germinal center–independent SHM. In particular, Warsame et al. showed evidence of ongoing mutations in micro dissected monocytoid B cells and expression of activation-induced cytidine deaminase (AID) which is required for SHM [35]. However, according to the BASELINE method, we found a positive selection only in 4 cases, whereas in the remaining cases, there was an unspecific antigenic stimulation that might reflect the necessity of preserving the integrity of BCR enabling the neoplastic cells to avoid apoptotic death [27]. Thus, this finding implies that the presence of BCR itself is necessary to generate a survival signal in the malignant cells.

Taking into account all of the above findings, the obvious conclusion is that environmentally encountered antigen plays at least some part in the maintenance of neoplastic phenotype in NMZL. Hence, immunogenic and functional evidence supports a role for antigen in the natural history of a subset of NMZL. However, the timing and duration of antigen interactions and their relevance for evolution of the disease remain elusive.

In addition, oncogenic events contribute to lymphoma growth and progression and may represent the first step of malignant transformation as demonstrated in recent genomic studies. Consistent with the physiological involvement of *NOTCH*, NF-κB, B cell receptor, and toll-like receptor signalling in the differentiation of mature B cells into the marginal zone B cells, many oncogenic mutations of genes involved in these pathways have been identified in MZL [36, 37]. In particular, although the NMZL genetic signature largely overlaps with SMNL, somatic coding-sequence mutations and deletions of the receptor-type tyrosine phosphatase gene *PTPRD* have been identified as a molecular feature of NMZL among indolent B cell tumors [2].

Interestingly, a subset of our cases (28.5%) did not carry SHM. The existence of unmutated IGHV genes could mean that the transformation leading to NMZL does not target exclusively post-germinal center B cells that bear SHM and have been submitted to T-dependent antigen selection. Conversely, U-NMZL may represent a subgroup not arising from post-germinal center B cells with a different pathogenesis which originates from a cell that has maturated outside of the germinal center and still maintains a naive-like epigenetic signature. Indeed, the possible presence of both virgin B cells and hypermutated B cells in NMZL suggests different modalities for the recruitment of B cells in the marginal zone [38]. Thus, in accordance with previous studies, the observed pattern of V_H_ mutations suggests that NMZL may originate from different subsets of marginal zone B cells: the naive B cells that express unmutated V_H_ genes and memory B cells characterized by somatic mutations [34].

The molecular heterogeneity that characterizes NMZL may thus reflect two molecular subtypes of the disease with two different cells of origin. The analysis of IGHV genes of other B cell lymphomas, including chronic lymphocytic leukemia (CLL), splenic marginal zone lymphoma (SMZL), and mantle cell lymphoma (MCL), has also revealed an unexpected heterogeneity in mutational status [39]. This heterogeneity has also been related to prognosis particularly in CLL, in which IGHV sequence analysis has become widely used for the purpose of prognostication [40, 41]. No international prognostic scoring system is available for NMZL and the value of biomarkers in NMZL remains unclear because of the small size of the series, heterogeneity of treatment, and lack of prospective clinical trials [17–45]. According to our knowledge, this is the first report which points out at the mutational status of the immunoglobulin genes as a prognostic biomarker for stratifying NMZL patients. In fact, cases characterized by unmutated immunoglobulin genes show a more aggressive clinical course. In particular, the disease-specific survival and the progression-free survival were significantly different between cases with mutated or unmutated IGHV genes. However, due to a limited number of cases, our results need to be confirmed in additional series of patients, possibly in prospective clinical trials, before applied in clinical practice.

On the other hand, we did not detect a correlation between the usage of a specific VH gene with survival probability. Further studies with larger populations will be needed to determine whether there is an association between VH gene usage and prognosis and whether there is a parallel or not with CLL.

In summary, we have shown that NMZL cells show a biased usage of IGHV genes in favor of specific segments and the role of antigenic stimulation in the aetiology of NMZL by maintaining BCR integrity. In addition, the postulated normal counterpart of this lymphoma consists of specific B lymphocyte subsets, with unmutated and mutated IGHV genes, expanding the overlap among small B cell lymphomas in terms of cell of origin and clinical outcome.

## Electronic supplementary material

Supplementary Fig1: **Antigen selection analysis.** The histograms (BASELINE) provide a visualization of selection pressure for complementarity-determining regions (CDRs) (red lines) and framework regions (FRs) (blue lines) in the heavy chains. Example of positive selection in the CDRs and negative selection in the FRs (A). Example of negative selection in the FRs and CDRs (B). (PNG 616 kb)

High resolution image (TIF 248 kb)

Supplementary Table 1: **Detailed results of high-throughput sequencing analysis of IGHV gene repertoire in 28 NMZL. (PNG 1599 kb)**

High resolution image (TIF 334 kb)

Supplementary Table 2: **Selection pressure using Bayesian estimation of antigen–driven selection.** BASELINE chart showing selection pressure by antigens in 4 patients (highlighted in yellow). In the remaining cases, except one, it is shown only a negative selection in the FRWS. (PNG 2407 kb)

High resolution image (TIF 141 kb)

Supplementary Table 3: **Multivariate Cox survival analysis.** The analysis points out that IGHV mutational status is a statistically significant prognostic indicator in both PFS and DSS analyses (*p* =0.002 and *p*=0.005 respectively). (PNG 572 kb)

High resolution image (TIF 237 kb)
