# Supplementary figures and images for: IGHV mutational status of nodal marginal zone lymphoma by NGS reveals distinct pathogenic pathways with different prognostic implications
Source: Virchows Arch. 2019 Dec 4;477(1):143–50. doi: 10.1007/s00428-019-02712-8 (PMC7320062; doi:10.1007/s00428-019-02712-8)

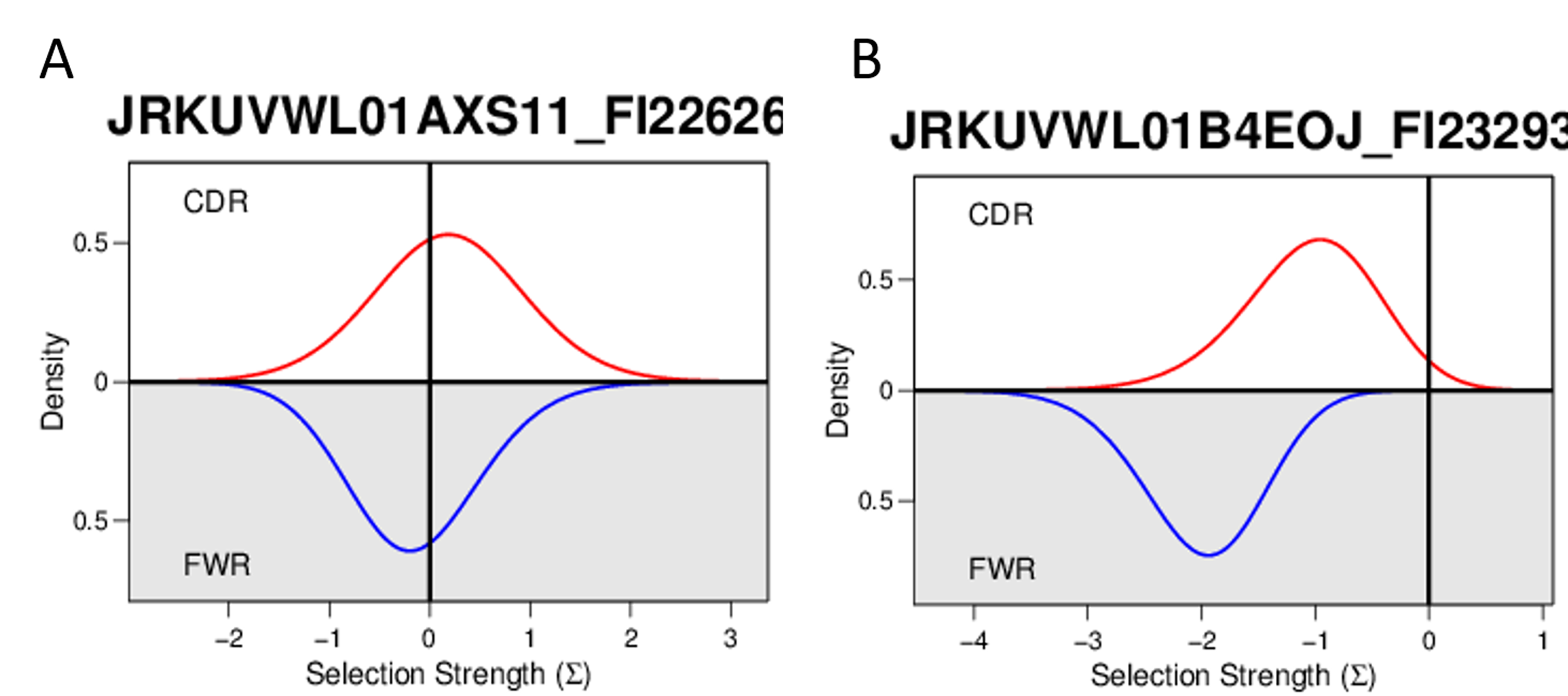

Supplement: Supplementary file 1 — Antigen selection analysis. The histograms (BASELINE) provide a visualization of selection pressure for complementarity-determining regions (CDRs) (red lines) and framework regions (FRs) (blue lines) in the heavy chains. Example of positive selection in the CDRs and negative selection in the FRs (A). Example of negative selection in the FRs and CDRs (B). (PNG 616 kb) [file 428_2019_2712_Fig4_ESM.png]

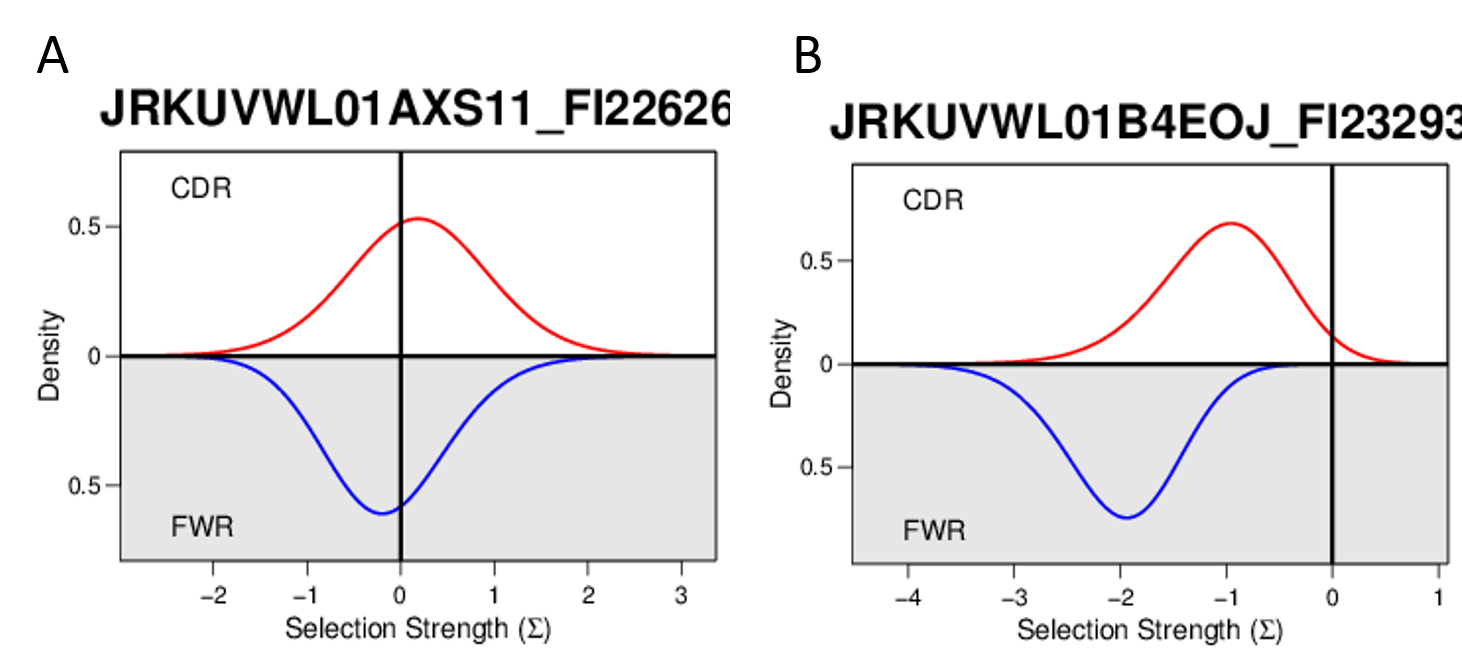

Supplement: Supplementary file 2 — High resolution image (TIF 248 kb) [file 428_2019_2712_MOESM1_ESM.tif]

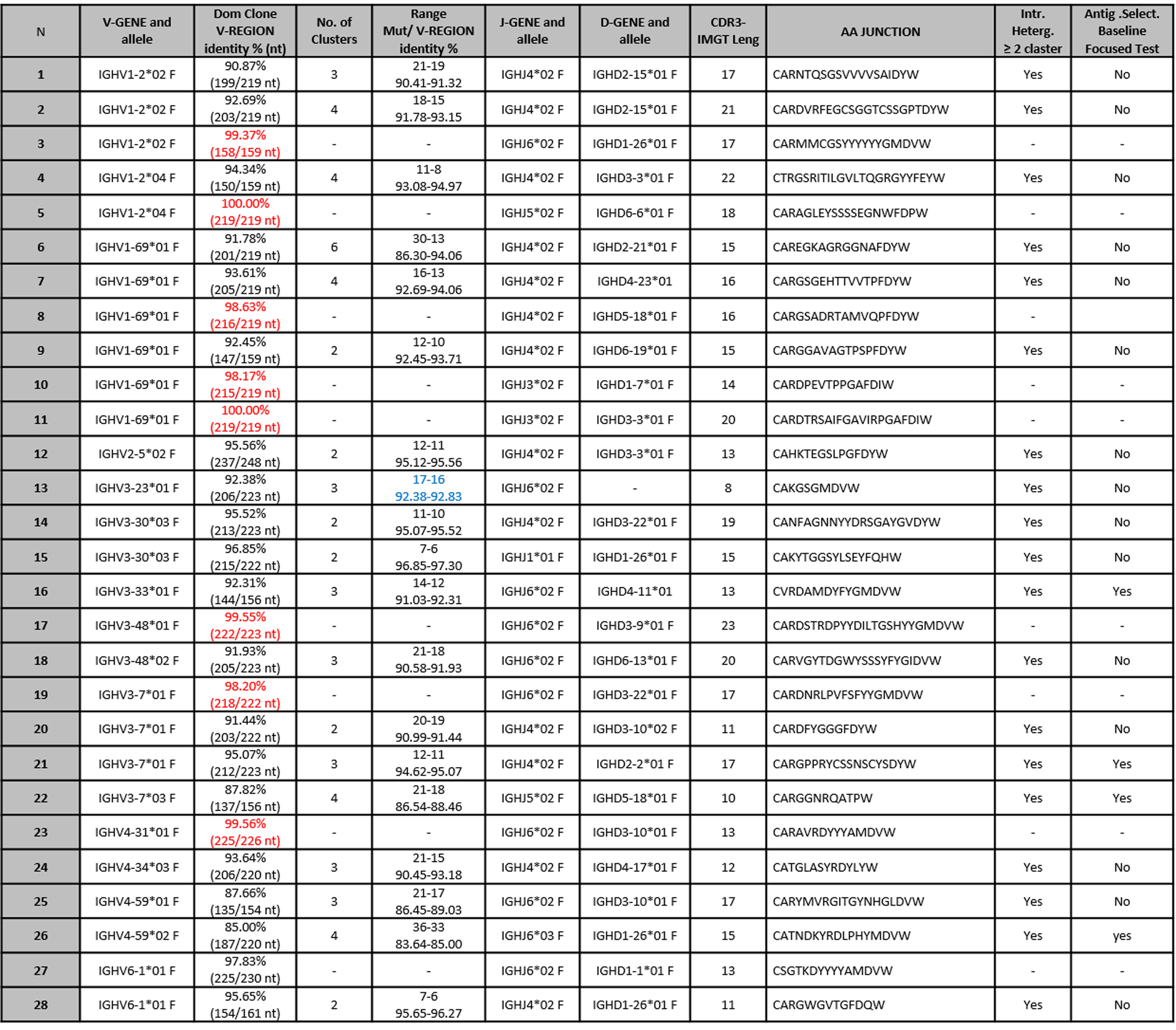

Supplement: Supplementary file 3 — Detailed results of high-throughput sequencing analysis of IGHV gene repertoire in 28 NMZL. (PNG 1599 kb) [file 428_2019_2712_Fig5_ESM.png]

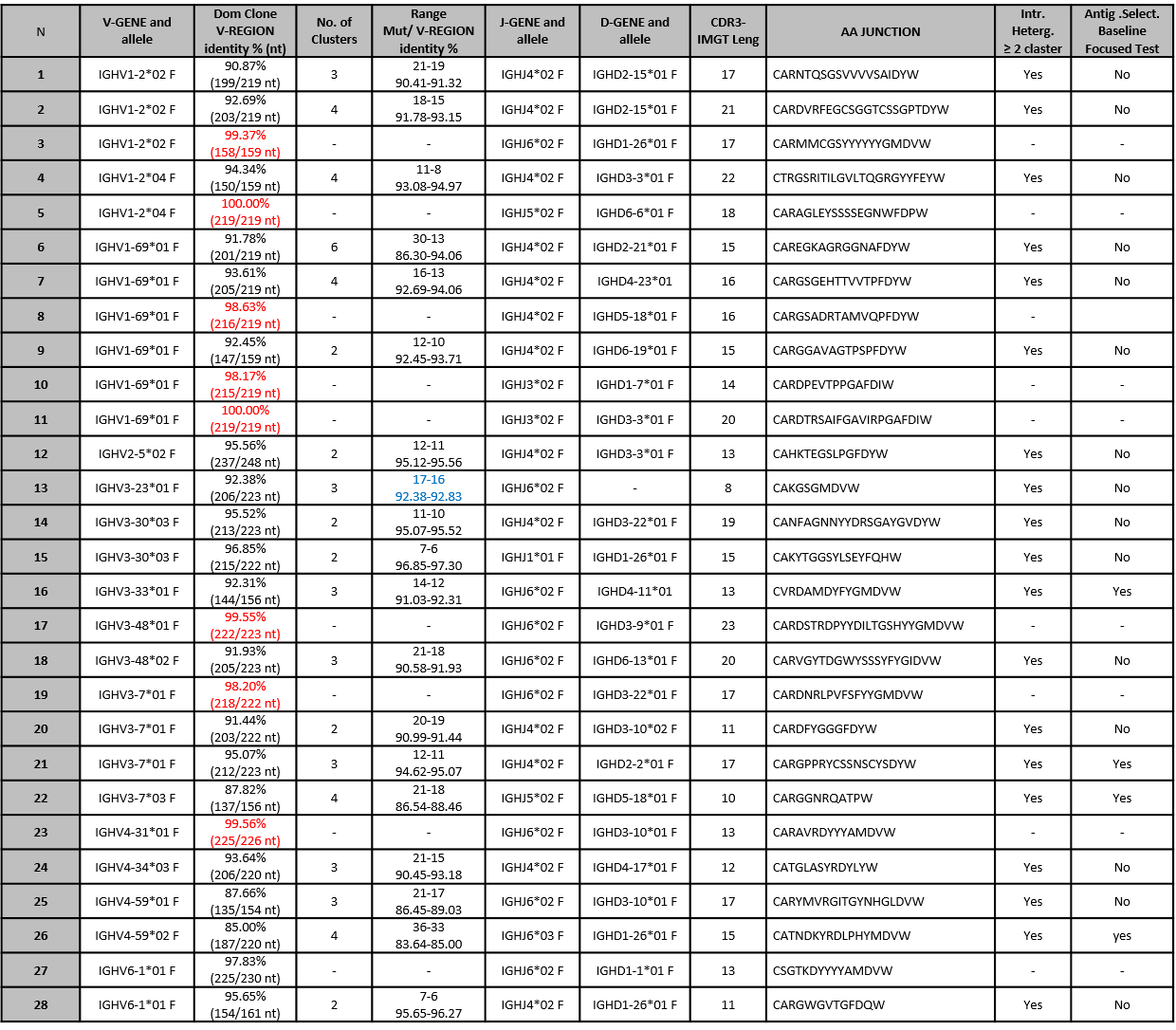

Supplement: Supplementary file 4 — High resolution image (TIF 334 kb) [file 428_2019_2712_MOESM2_ESM.tif]

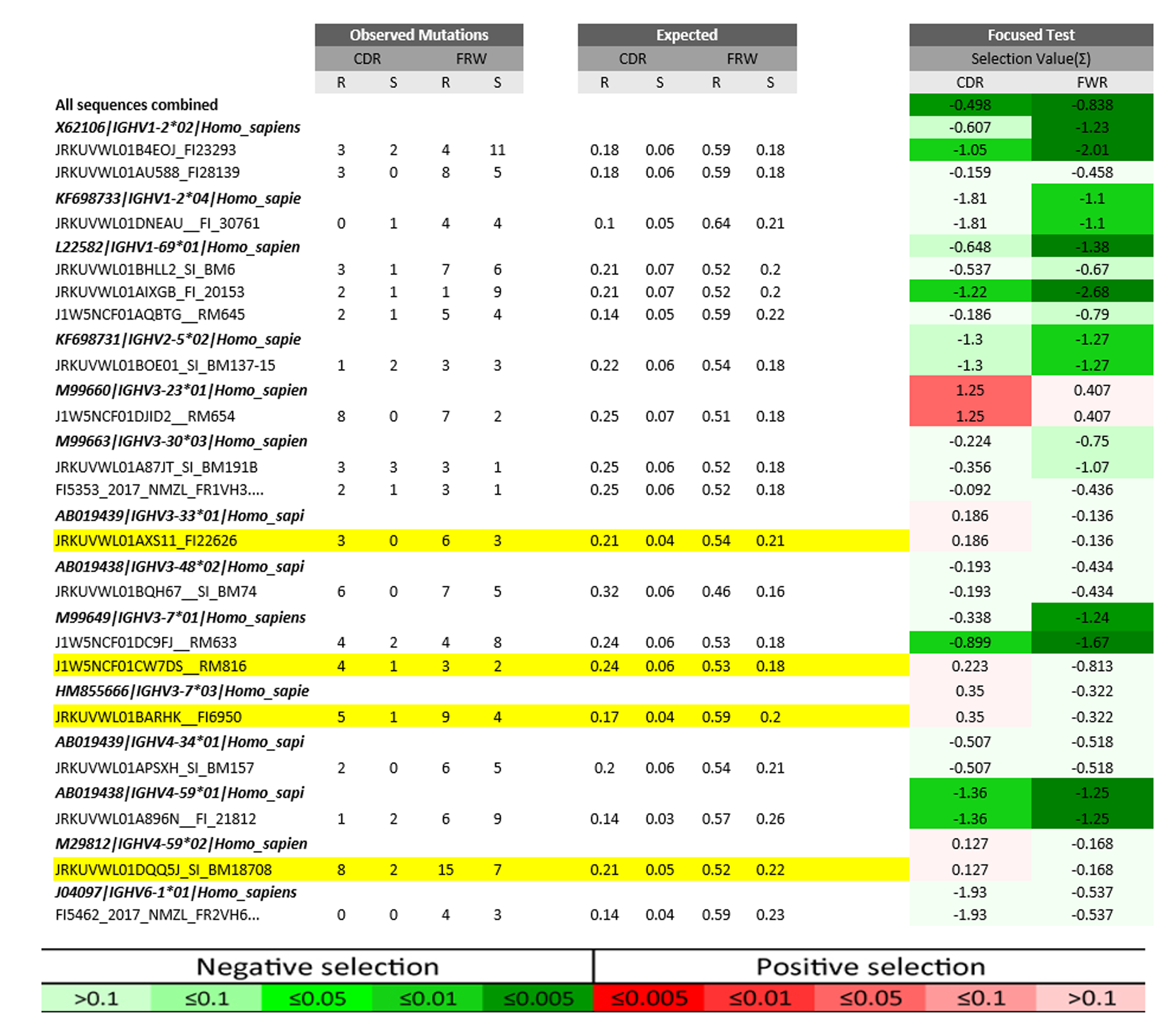

Supplement: Supplementary file 5 — Selection pressure using Bayesian estimation of antigen–driven selection. BASELINE chart showing selection pressure by antigens in 4 patients (highlighted in yellow). In the remaining cases, except one, it is shown only a negative selection in the FRWS. (PNG 2407 kb) [file 428_2019_2712_Fig6_ESM.png]

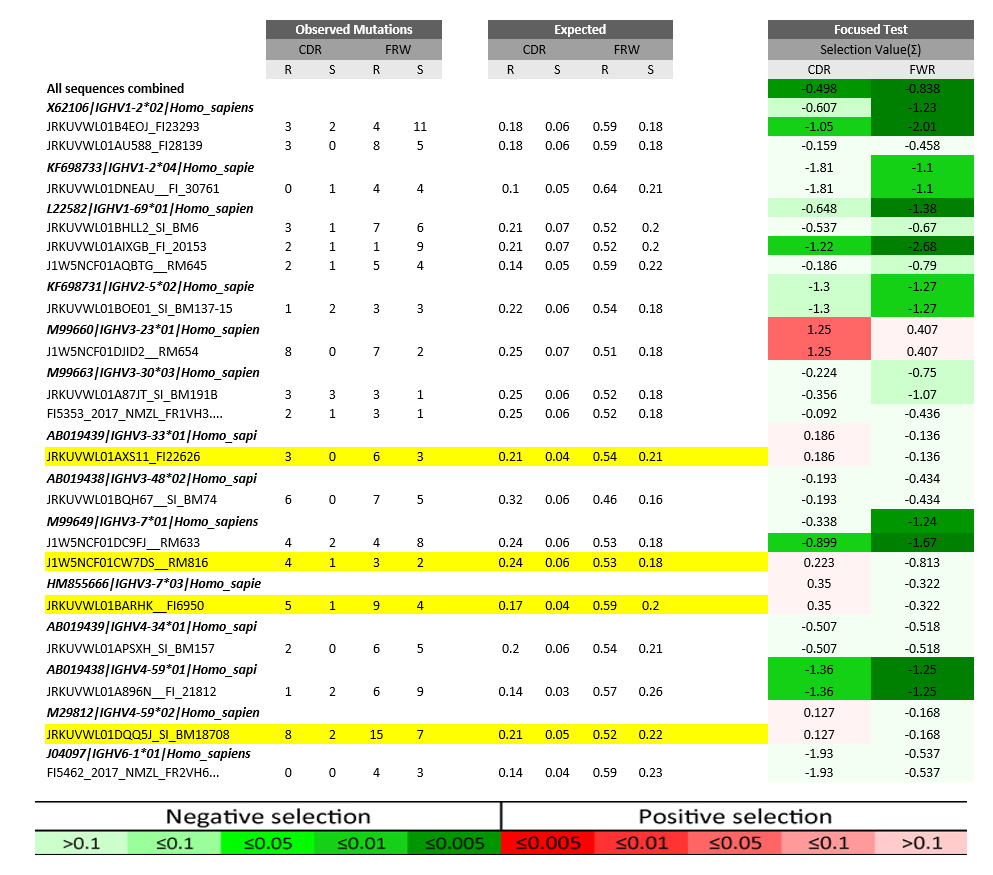

Supplement: Supplementary file 6 — High resolution image (TIF 141 kb) [file 428_2019_2712_MOESM3_ESM.tif]

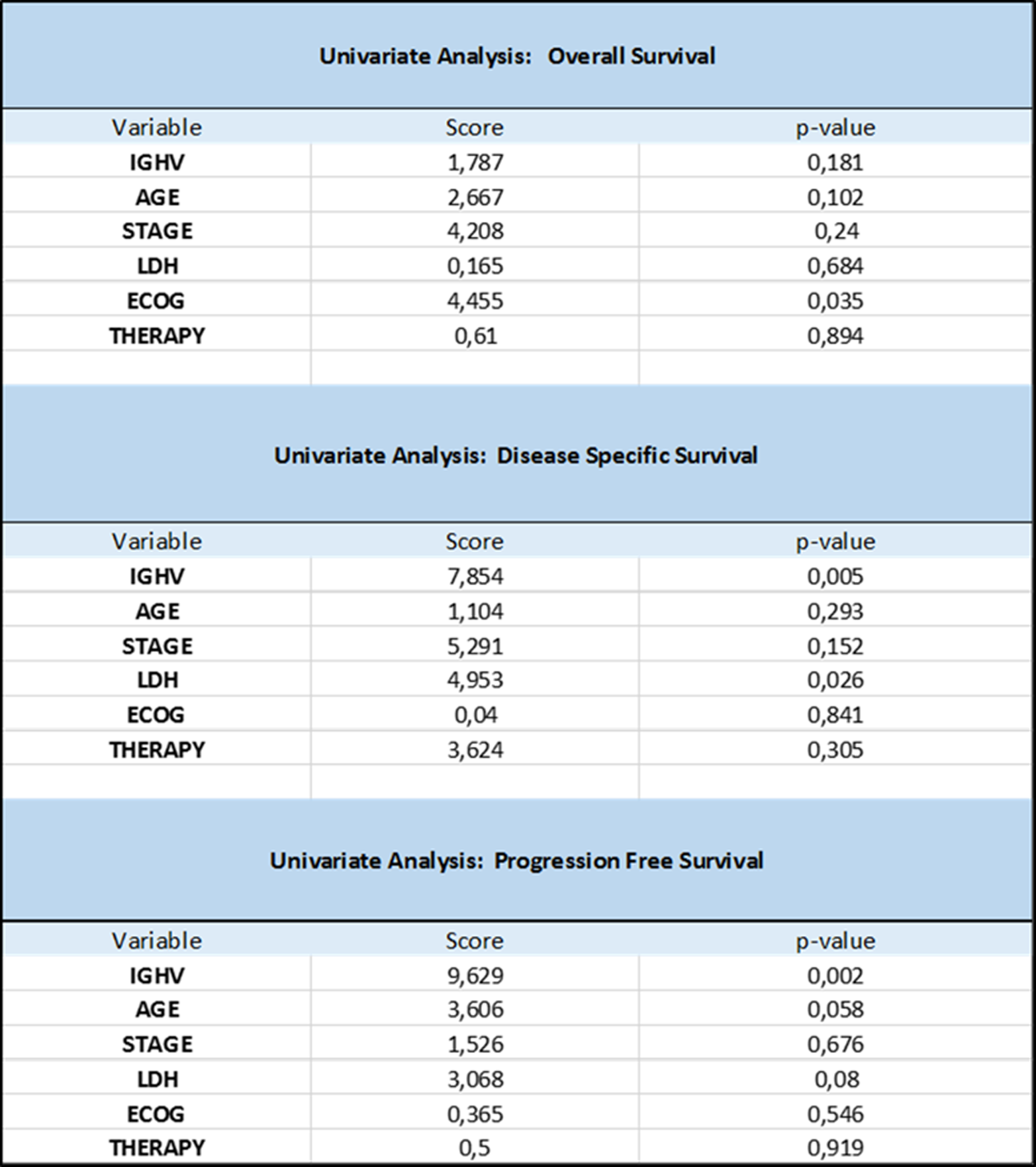

Supplement: Supplementary file 7 — Multivariate Cox survival analysis. The analysis points out that IGHV mutational status is a statistically significant prognostic indicator in both PFS and DSS analyses (p =0.002 and p=0.005 respectively). (PNG 572 kb) [file 428_2019_2712_Fig7_ESM.png]

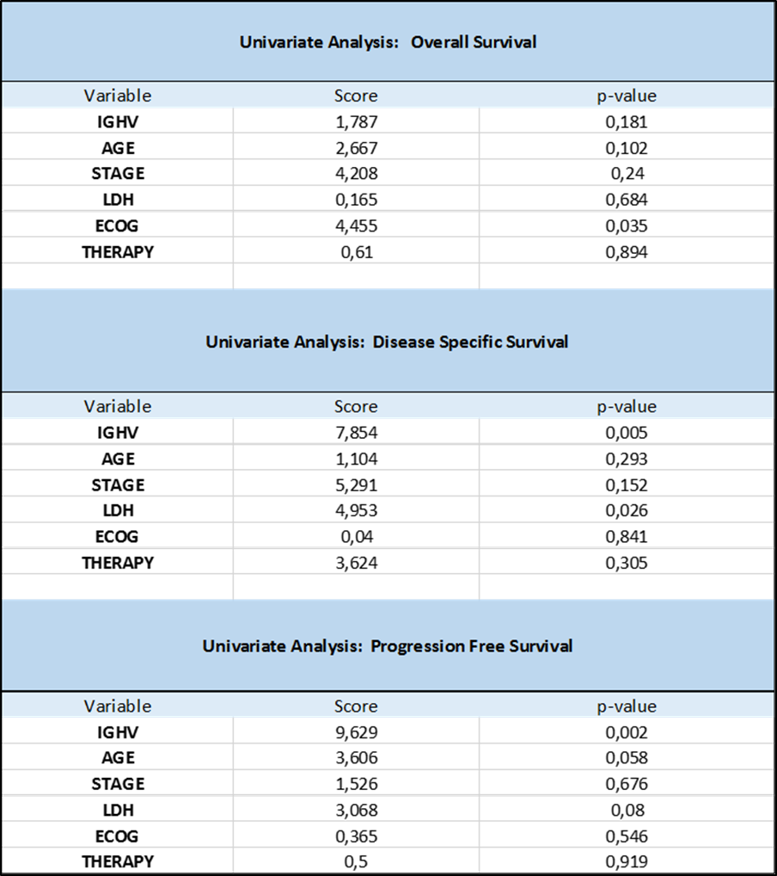

Supplement: Supplementary file 8 — High resolution image (TIF 237 kb) [file 428_2019_2712_MOESM4_ESM.tif]
